# The Father in Youth Baseball: A Self-Determination Theory Approach

**DOI:** 10.3390/ijerph18094587

**Published:** 2021-04-26

**Authors:** Manuel De La Cruz, Jorge Zamarripa, Isabel Castillo

**Affiliations:** 1 Licenciatura en Entrenamiento Deportivo, Universidad Estatal de Sonora, Hermosillo 83100, Mexico; manueldelacruz@ues.mx; 2 Facultad de Organizacion Deportiva, Universidad Autonoma de Nuevo Leon, San Nicolas de los Garza 66455, Mexico; 3 Department of Social Psychology, University of Valencia, 46010 Valencia, Spain; Isabel.Castillo@uv.es

**Keywords:** self-determination theory, basic psychological needs, life aspirations, intentions, youth baseball

## Abstract

This study based on the self-determination theory aims to examine the relationship among the aspirations that fathers have about their children’s youth baseball practice, the children’s basic psychological needs (satisfaction and frustration), and their intentions to either continue or drop out of baseball practice in a sample of children from Hermosillo, Mexico. A cross-sectional study was carried out involving 533 fathers (M = 44.30, SD = 5.18) and 533 children (M = 13.09, SD = 1.68). The results showed that the intrinsic aspirations of fathers were positively correlated to the satisfaction of the children’s psychological needs, whereas the extrinsic aspirations of fathers were positively correlated with the frustration of the children’s psychological needs. Satisfaction of basic psychological needs was positively correlated with the intention to continue and negatively correlated with dropout; on the contrary, frustration of basic psychological needs was negatively correlated with the intention to continue and positively with dropout. In conclusion, the fathers’ pursuit of intrinsic aspirations for their children in the youth baseball context satisfies the children’s basic psychological needs, and in turn, their intention to continue practicing increases. Conversely, when a father pursues extrinsic aspirations for his son in youth baseball, the child will feel his basic psychological needs frustrated, and he will have a greater intention to drop out. Overall, this study extends the existing sport-scientific literature by confirming the impact of parents’ aspirations on their children’s basic psychological needs and intention to continue being baseball players.

## 1. Introduction

Many factors influence people’s behavior toward sports [1]. Among these factors is family influence, which relies on developing interests and skills that reinforce active behavior [2]. Psychological theories assume that the values, the norms, and the behavioral model are transmitted through parent–child relationships [3]. From this perspective, physical–sports socialization is a modeling process in which a group of significant others, such as parents, constitute an important social influence model for people [4], and where the father plays a more dominant role than the mother in shaping their children’s perceptions and affective outcomes [5,6,7].

The goal content theory, which is a subtheory of self-determination theory (SDT) [8,9], distinguishes between intrinsic and extrinsic aspirations. Intrinsic aspirations are a congruent expression of a human being’s natural desire for growth and self-realization, and include affiliation (close relationships), which refers to having satisfactory relationships with family and friends; community involvement (community contributions), which means improving the world through activism and generativity; physical condition (health), namely feeling healthy and free of ailments; and self-acceptance (personal growth) as regards the achievement of psychological growth, autonomy, and self-esteem [10,11]. Extrinsic aspirations are those that depend on the reactions from others. Usually, people with these kinds of aspirations are involved in an activity as a means to achieve another purpose, including financial success (wealth), which refers to being affluent and materially successful; social recognition (fame), which means being famous, well-known, and admired; and attractive appearance (image), namely claiming to be attractive in terms of body image, clothing, and fashion [10,11].

In contemporary society, the achievements of children are seen as a reflection of good parenting skills [5]. This means that parents strive to prove themselves through the achievements of their children, believing that, thanks to these achievements, their child’s life will be happy and successful [12]. Considering this, some parents are involved in children’s sports to satisfy their own aspirations and indirectly experience their children’s achievements [13]. The money from contracts, the recognition, and the fame that can be achieved by becoming a professional athlete may seem attractive to a father, and by being responsible for raising his child, he could decide to turn him into a professional athlete [14]. In other words, many of these parents pursue certain aspirations for their children’s sport participation, and it may be of interest to know what effects these parental aspirations may have on how their children feel during sport participation and what their children’s intentions are about their sporting future (i.e., whether they want to continue or rather want to drop out of sport).

The basic psychological needs (BPN) theory, another subtheory of SDT [8,15], postulates that when individuals interact with their environment, they need to feel autonomous (feeling that they are the initiators of their own actions), competent (feeling capable of interacting effectively with the environment), and related to others (feeling connected and respected by others, i.e., a feeling of belonging to a group). These three BPN (autonomy, competence, and relatedness) are essential to the growth, integrity, and well-being, and significant figures (e.g., coaches, parents, teammates, etc.) have the potential to influence athletes’ participation through their satisfaction or frustration. There is a large body of research in the sport domain that has used BPN as the mediational mechanism in the relationship between the social context of young players, predominantly coaches’ behaviors (e.g., motivational climate, interpersonal styles), and different athletes’ outcomes, such as indicators of well- and ill-being, quality of sport experience, and future intention to continue being physically active or drop out of sport [16,17,18,19,20,21,22,23,24,25,26]. However, to date, few studies have analyzed what kind of aspirations parents pursue in their children’s sports practice and the effect these parental aspirations have on their children’s thoughts, cognitions, and behaviors through their BPN satisfaction or frustration, taking into account that the information was collected exclusively by asking the children, without collecting information provided by the father [e.g., 27,28]. For example, the study by Wouters et al. [27] revealed a positive correlation between the intrinsic aspirations of the parents (provided by the children) and their children’s BPN satisfaction, finding no relationship with extrinsic aspirations of the parents. Similarly, Chew and Wang [28] found that both types of aspirations were positively correlated with the need for competence in sport. In addition, the perception of autonomy support from the parents was positively associated with the satisfaction of the BPN, and it was positively correlated with intrinsic aspirations and well-being.

The present study aims to enhance our understanding of parental aspirations’ influence on adolescents’ outcomes, using the SDT framework and considering both parents’ and children’s perceptions. That is, the dyadic parent–child relationship will be considered.

Sonora belongs to the Northwestern region of Mexico, which is the one region that has contributed the most players to the Mexican Baseball League [29,30]. This might cause parents to have extrinsic aspirations related to fame, wealth, and self-image over intrinsic aspirations for their children. In this line, several studies based on the SDT have been carried out [8,15,31] focusing on parents and their impact on the child’s psychological development and well-being. However, no studies so far are known to have addressed the relationship between the aspirations of parents and their children’s baseball practice; therefore, this study aims to examine the relationship among the aspirations of fathers about their children’s baseball practice, their children’s basic psychological needs (satisfaction and frustration), and their children’s intention to either continue or drop out from baseball practice in a sample of children who play youth baseball in Hermosillo, Sonora.

**Figure 1:**
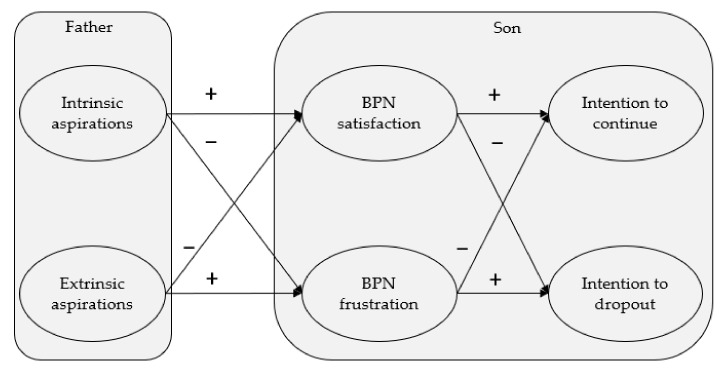
Hypothesized model of the relationships between fathers’ aspirations and the children’s basic psychological needs (satisfaction and frustration) and future intentions.

To reach our goal, four hypotheses were tested. H1: The intrinsic aspirations from fathers about their children’s baseball practice will be positively associated with the satisfaction of the children’s BPN and negatively associated with their frustrated needs. H2: The extrinsic aspirations from fathers about their children’s baseball practice will be negatively correlated with the satisfaction of their children’s BPN and positively correlated with their frustrated needs. H3: The satisfaction of the children’s BPN will be positively associated with the intention to continue playing baseball and negatively associated with the intention to dropping out baseball. H4: The frustration of the children’s BPN will be negatively correlated with the intention to continue baseball practice and positively correlated with the intention of dropping out from baseball practice (see Figure 1).

## 2. Materials and Methods

### 2.1. Participants

Sampling and recruitment were by convenience, provided that the inclusion criteria were met. Since the project contemplates the testing of a theoretical model through the analysis of structural equation models, the sample size was determined using the following formula: 1/2 × *q* × (*q* + 1), where *q* is the number of observed variables.

Inclusion criteria were that the child was affiliated to a league and the age range was between 10 and 16 years. To homogenize the study, the father was contacted, as according to the literature, it is the father who exerts the greatest influence in the sporting context e.g., [7]. No parents with more than one child in the league were detected. In order to be able to match the father’s questionnaire with the corresponding child, both were asked for the same code, consisting of adding the initials of the child’s first and last name and the date of birth.

Out of a total of 538 parents (all parents whose children were enrolled in the league), 1% (*n* = 5) chose not to participate in the study. The final sample size was five hundred and thirty-three fathers ranging from 31 to 68 years old (*M* = 44.30, *SD* = 5.18) and their sons (533 male baseball players) aged between 10 and 16 years old (*M* = 13.09, *SD* =1.68) participated in this study. The full sample of 1066 participants was represented in the hypothesized model. The players belonged to nine youth baseball leagues from Hermosillo, Mexico.

### 2.2. Instruments

Intrinsic and extrinsic aspirations of the father were measured using a Spanish translation of the *Aspiration Index* [10], adapting the phrasing of the items so that it could be answered by the father. This instrument measures the importance, probability, and achievement of two groups of aspirations that pursue intrinsic and extrinsic aspirations. Intrinsic aspirations include personal growth, close relationships, community contributions, and health; whereas extrinsic aspirations encompass wealth, fame, and image; each of these aspirations is measured through five items. For this study, the composite value of intrinsic and extrinsic aspirations was used by calculating the mean of the variables that make up each of the factors. The instrument was headed by the phrase “How important is it to you…”. An example of an intrinsic aspiration item is “…that your son grows up and learns new skills”; and an example of an extrinsic aspiration item is “…that your son becomes a very wealthy person”. Fathers responded on a Likert scale ranging from 1 (“*not important at all*”), 3 (“*moderately important*”), to 7 (“*very important*”). The reliability of this instrument has been shown by previous studies, reporting Cronbach’s alpha coefficients between 0.80 and 0.96 for intrinsic aspirations and 0.71 and 0.92 for extrinsic aspirations [28,32,33,34].

Satisfaction and frustration of the son’s basic psychological needs. The Spanish version of the *Basic Psychological Need Satisfaction and Frustration Scale* [35] was used and adapted to the baseball context. This scale consists of 24 items that measure the satisfaction or frustration of six factors with four items each, which are satisfaction of autonomy, competence, and relatedness; and frustration of autonomy, competence, and relatedness. The instrument was headed by the phrase “During my baseball practice…”. An example of a satisfied need item is “I feel skilled in the activities I do”; and an example of a frustrated need item is “I feel unsure about my abilities”. The responses are collected on a Likert scale ranging from 1 (“*completely false*”) to 5 (“*completely true*”). The factorial structure and the reliability of this instrument have been shown in other studies, reporting Cronbach’s alpha coefficients between 0.81 and 0.90 for needs satisfaction and 0.83 and 0.86 for needs frustration [36,37,38].

Son’s intention to continue and drop out from baseball. The *Future Intention to Practice Baseball Scale* adapted to baseball was used to assess the intention to continue [39] and the intention to drop out from baseball practice [40]. The intention to continue subscale consists of three items. An example item is, “I plan to play baseball next season”. The responses were collected on a Likert scale ranging from 1 (“*It says nothing about me*”) to 7 (“*It completely describes me*”). Previous research has verified the unifactorial validity of the scale, as well as its reliability, showing Cronbach’s alpha coefficients between 0.90 and 0.95 [39,40,41]. The dropping out intention subscale consists of five items, and the responses are collected on a Likert scale ranging from 1 (“*completely disagree*”) to 7 (“*strongly agree*”). An example of item is, “I’m seriously thinking about dropping out from baseball”. Evidence for the unidimensional structure and reliability has been provided by previous research, with Cronbach’s alpha ranging from 0.88 to 0.91 [21,42,43,44,45].

### 2.3. Procedure

This research was conducted in accordance with international ethical standards, which are consistent with the guidelines of the American Psychological Association (APA) and the Declaration of Helsinki. The study was registered and approved by the ethics review committee of the research coordinating office of the Faculty of Sports Organization of the Autonomous University of Nuevo Leon (No. REPRIN-FOD-81).

Authorization was requested from the baseball league to access the facilities, where parents were summoned to learn the purpose of the study, accept the request for their participation in the study, answer the instrument, and sign the informed consent for their children to fill the instruments in a separate session. The instruments were translated into Mexican Spanish using the back-translation method. The translation was carried out by a professional translation agency hired by the team in charge of the study. For its adaptation to the baseball framework, a group of experts (composed of two doctors with previous experience validating psychological instruments, a sports coach, and a translator specialized in the area of physical education and sports) discussed the discrepancies in the translation until a first version of the instrument in Mexican Spanish was agreed upon. This version was translated back into English by a professional translation agency different to the one previously hired, and then, both versions of the instrument were compared: the original source and the translation. The inconsistencies of each version were analyzed again, and changes were made to facilitate the comprehension of the items, attaining a final version for each of the scales. This version was presented as a pilot to verify that each item was understandable; according to the pilot results, no comprehension issues were found, showing Cronbach’s alpha coefficients of 0.93 for extrinsic aspirations and 0.75 for intrinsic aspirations (*n* = 46).

### 2.4. Data Analysis

No missing data were detected. Descriptive, normality, and reliability analyses were performed for the scales using the SPSS Statistics V.24.0 statistical software (IBM Corp., Armonk, NY, USA). Data distribution will be considered normal when the skewness and kurtosis indices range between −1 and 1 [46,47,48]. The internal consistency of each scale and subscale of the instruments was evaluated using Cronbach’s alpha [49], Composite Reliability (CR), and Average Variance Extracted (AVE) based on a confirmatory factor analysis. Overall, alpha values greater than 0.70 for Cronbach’s alpha, and McDonald’s omega coefficients will be considered indicative of good and acceptable reliability [50]. AVE reflects the total variance of the indicators collected by the latent construct, and it is suggested that its value should exceed 0.50 [51,52].

Secondly, confirmatory factor analyses (CFA) of the study instruments were performed using the LISREL 8.80 software [53]. For the Aspiration Index, a model of seven first-level factors was tested: four factors (personal growth, close relationships, community contributions, and physical health) and three factors (wealth, fame, and image), out of which stem two latent exogenous variables of a higher order called intrinsic and extrinsic aspirations. For the Satisfaction and Frustration of Basic Psychological Needs Scale, a model of six first-level factors was tested: three factors comprised of satisfaction of autonomy, relatedness, and competence, and three more composed of frustration of autonomy, relatedness, and competence, which in turn form, in a second order, BPN satisfaction and frustration. Finally, a two-factor model called “intention to continue and intention to drop out” was tested for the Future Intention to Practice Baseball Scale. Since the observed variables are of the ordinal type, and to correct the possible lack of data normality, the polychoric correlation matrix and the asymptotic covariance matrix were used as input for the CFA; likewise, the estimation method used was Maximum Likelihood, because it works well with large size samples, even when the number of variables is high, and especially if the number of factors to retain is small [54].

Lastly, the total measurement model was tested, which is equivalent to a confirmatory factorial model, also testing the divergent validity of the latent variables. In addition, the hypothesized structural equation model was tested (see Figure 1) using latent variables. The polychoric correlation and the asymptotic covariance matrices were used as input for the analysis. The data were analyzed using the Maximum Likelihood analysis with LISREL 8.80 [53]. The model’s adequacy was analyzed through different fit indices: the chi-square value divided by its degrees of freedom (χ^2^/df), Non-Normed Fit Index (NNFI), Comparative Fit Index (CFI), Root Mean Square Error of Approximation (RMSEA), and Standardized Root Mean Square Residual (SRMR). A χ^2^/df ratio of less than 5 indicates a good model fit [55]. CFI and NNFI values above 0.90 indicate an acceptable fit [56]. For RMSEA and SRMR, values between 0.05 and 0.10 are considered acceptable (satisfactory: equal to or less than 0.08 [57]). We calculated the mean of the fathers’ aspirations and three psychological needs to assess the overall satisfaction and frustration of the three needs following the postulates of the SDT and the procedures used in previous studies [24,26,58].

## 3. Results

### 3.1. Descriptive and Reliability Analysis

Table 1 shows means, standard deviations, skewness, and reliabilities of all the study variables. Fathers’ responses showed that the value for intrinsic aspirations was above the mean value of the questionnaire, while extrinsic aspirations was under the mean value. Players’ responses showed that values for BPN satisfaction and intention to continue were above the mean, while BPN frustration and intention to drop out were nearly under or under the mean value. Likewise, it can be observed that skewness and kurtosis values do not follow a normal distribution (see Table 1).

The internal reliability coefficients were satisfactory for all the scales (see Table 1). When AVE is less than 0.50, but CR is higher than 0.60, the convergent validity of the variable can still be considered adequate [59].

The results of the higher-order CFA of the parental aspirations scale revealed satisfactory goodness-of-fit indices (χ^2^= 2240.32, *p* < 0.001, df = 552, χ^2^/df = 4.05, NNFI = 0.96, CFI = 0.97, RMSEA = 0.07, SRMR = 0.08). The model showed factor loadings ranging from 0.61 to 0.85 for intrinsic aspirations and factor loadings ranging from 0.56 to 0.98 for extrinsic aspirations. Regarding the Satisfaction and Frustration of the Basic Psychological Needs Scale, the goodness-of-fit indices of the two-factor model were satisfactory (χ^2^= 835.11, *p* < 0.001, df = 245, χ^2^/df = 3.40, NNFI = 0.97, CFI = 0.98, RMSEA = 0.06, SRMR = 0.08). The model in the first-level factors showed factor saturations ranging from 0.41 to 0.79 for BPN satisfaction, while BPN frustration showed factor saturations ranging from 0.68 to 0.83. Regarding the Future Intention to Practice Scale, a two-factor model was tested with satisfactory goodness-of-fit indices (χ^2^ = 55.22, *p* < 0.001, df = 19, χ^2^/df = 2.91, NNFI = 0.99, CFI = 0.99, RMSEA = 0.06, SRMR = 0.03). The two-factor model showed factor saturations ranging from 0.54 to 0.94 for the intention to continue playing baseball, and factor saturations ranging from 0.85 to 0.97 for the intention to drop out from baseball. The factor saturations of the scale items were statistically significant (*p* < 0.001); namely, each item constitutes a reliable indicator of the subscale of which it is part (see the Supplementary Materials for more details).

### 3.2. Correlation Analysis

As expected, a positive correlation was found between the intrinsic aspirations of the fathers and BPN satisfaction, and between the extrinsic aspirations of the fathers and BPN frustration. BPN satisfaction was positively associated with the intention to continue and negatively correlated with the intention to drop out. Conversely, BPN frustration was negatively associated with the intention to continue and positively correlated with the intention to drop out (see Table 2).

### 3.3. Measurement and Structural Equations Models

**Figure 2:**
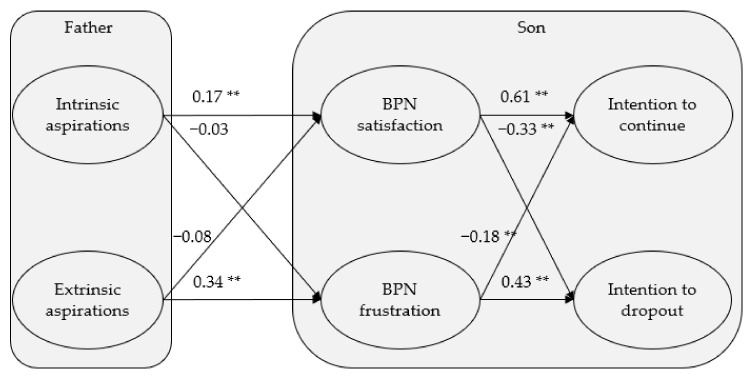
Standardized solution of the relationship model between parental aspirations and their children’s basic psychological needs and their future intentions. ** *p* < 0.01.

The measurement model presented acceptable fit indices: χ^2^ = 6796.03, df = 2129; *p* < 0.001; χ^2^/df = 3.19; NNFI = 0.94; CFI = 0.95; RMSEA = 0.06; SRMR = 0.07 confirming the divergent validity of latent variables [60]. The structural equation model also presented acceptable fit indices: χ^2^ = 6809.06, df = 2133, *p* < 0.001; χ^2^/df = 3.19; NNFI = 0.94; CFI = 0.95; RMSEA = 0.06, SRMR = 0.07. Figure 2 shows how the fathers’ intrinsic aspirations were significantly and positively correlated with their children’s BPN satisfaction, while the relationship between the fathers’ intrinsic aspirations and BPN frustration was not significant. In contrast, the fathers’ extrinsic aspirations were significantly and positively correlated with their children’s BPN frustration, and the relationship between extrinsic aspirations and BPN satisfaction was not statistically significant. BPN satisfaction was positively and significantly correlated with the intention to continue and negatively and significantly with the intention to drop out. Finally, BPN frustration was positively and significantly correlated with the intention to drop out and negatively and significantly correlated with the intention to continue.

In addition to the direct effects indicated, the model also showed indirect effects of intrinsic aspirations on the intention to continue (β = 0.11, *p* < 0.01) and the intention to drop out (β = −0.07, *p* < 0.01) through the BPN satisfaction. Moreover, extrinsic aspirations showed indirect effects on the intention to continue (β = −0.11, *p* < 0.01) and the intention to drop out (β = 0.17, *p* < 0.01) through the BPN frustration. As a whole, the model predicted 3.1% of the variance of BPN satisfaction, 11.2% of the variance of BPN frustration, 52.1% of the variance of intention to continue playing baseball, and 43.5% of the variance of intention to drop out from baseball in the future.

## 4. Discussion

The purpose of this work based on the SDT [8] was to test a model postulating that the fathers’ aspirations would be associated with the satisfaction or frustration of their children’s BPN, which would lead to a greater intention either to continue or drop out from baseball practice. In general, the results supported the hypothesized model, emphasizing the importance of the fathers’ aspirations with respect to their children, given that these aspirations have either positive or negative implications on their sons’ baseball practice. While other studies have asked children about the aspirations they believe their parents have regarding their sports practice, in this study, we have directly asked parents about their aspirations for their children and children about their satisfaction or frustration of their basic psychological needs, as well as about their future intention to practice.

With respect to the first hypothesis (H1) of the proposed model, results revealed that the intrinsic aspirations of the fathers were positively correlated to BPN satisfaction; however, no relationship was found between the fathers’ intrinsic aspirations and BPN frustration, and therefore, our hypothesis H1 is rejected. However, this negative relationship was significant at the correlation of variables level. This is when the father has intrinsic aspirations for his child to play baseball so that he obtains personal growth, the feeling of contributing to improve society, close relationships that are of great value to him, and good physical health; in these circumstances, the child will become satisfied with his baseball skills, he will feel that there is congruence between what he wants to do and what he actually does, and he will feel appreciated by others in baseball. In addition, when the father has such intrinsic aspirations for his son in baseball, the child will not feel frustrated in relation to the competence, autonomy, and relatedness that he experiences when playing baseball. Our results are in line with the previous results obtained by Wouters et al. [27] with a sample of college students. These authors discovered that when children perceived that their parents promoted intrinsic aspirations, they tended to satisfy their BPN, and no significant relationship with BPN satisfaction was found when parents promoted extrinsic aspirations. Likewise, they are in line with the findings of Chew and Wang [28], who observed that intrinsic aspirations were positively correlated with the need for competence in sport. In summary, our results are in line with what has been previously stated about the Goal Contents Theory, which says that people who follow intrinsic aspirations for their inherent value to human beings do tend to satisfy their BPN [10,11].

With respect to the second hypothesis (H2) of the proposed model, results revealed that fathers’ extrinsic aspirations were positively correlated with BPN frustration and did not show a significant relationship with BPN satisfaction; therefore, our second hypothesis is rejected. The results previously described are in accordance with the Goal Content Theory premise that when people, in this case fathers, pursue extrinsic aspirations for their children, the latter will tend to have their BPN frustrated, understanding that fathers make their sons practice youth baseball as a means to achieve a totally different end from the main objective of this sport and rather seeking approval from other people [10,11]. In other words, children whose parents expect them to achieve financial success (wealth) through baseball, to become recognized and admired by other people (fame), and to look attractive using the best sportswear (image) will have a feeling of frustration of autonomy, they will feel that perhaps baseball is not what they want to practice; they will feel frustration of relatedness—in other words, they will not feel appreciated by others; and they will feel their competence need frustrated by not fulfilling their fathers’ aspirations.

With respect to the third hypothesis (H3) of the proposed model, results revealed that BPN satisfaction was positively correlated with the intention to continue playing baseball. These results are in line with the BPNT, which postulates that athletes who satisfy their BPN will tend to continue practicing sport [8,15]. There are studies that confirm this association [16,17,18,19,20,21,26]. A 19-month study [19] found that athletes with higher levels of BPN satisfaction continued practicing sports, in contrast to those with low satisfaction who drop out of sports practice. Likewise, our results have shown that BPN satisfaction was negatively correlated with the intention to drop out from future baseball practice. This is aligned with the findings of García-Calvo et al. [17] with a sample of soccer players, where those who dropped out from sport practice had significantly lower levels of satisfaction of their BPN compared to those players who continued practicing soccer. Similarly, our findings are in line with Quested et al.’s [21], which supported the relevance of BPN in explaining sports dropout across five European countries. BPN satisfaction predicted enjoyment, which in turn negatively predicted the intention to drop out from youth sports practice. In addition, González et al. [24] found a negative association between BPN satisfaction and burnout, the latter being positively related to sports dropout [22,25]. These results are consistent with the SDT, which states that BPN satisfaction is negatively correlated to the intention to drop out from sports practice [8,15]. In sum, when the child feels that he is playing baseball because it is what he wants to do, feels competent in learning new skills and is not based on winning or losing, and an environment is created where the child feels appreciated by the parent, the child will have the intention to continue playing and will not want to drop out from baseball. Overall, these results support hypothesis H3.

Finally, our results confirm the fourth hypothesis (H4) by showing us that BPN frustration was significantly and positively correlated with the intention to drop out from baseball practice and significantly and negatively with the intention to continue. These results are aligned with those shared by Castillo et al. [23] and González et al. [24] in terms of youth sports, as well as Pulido et al. [61], who found a positive relationship between BPN frustration and burnout, which in turn was positively correlated with the intention to dropout [25]. It is worth noting that these results confirm the SDT postulate that suggests that when the athletes’ BPN are frustrated, they will have the intention to drop out from sports [8,15]. In other words, when the child plays baseball even though he does not want to, feels that he is not competent to fulfill his father’s aspirations, and an environment is created in which he thinks that his father is only evaluating him, he will feel unappreciated and will intend to drop out from baseball.

One possible explanation as to why parents of our sample erroneously pursue extrinsic objectives comes from the possibility that their children will become professional athletes, despite the few opportunities that exist to achieve this. It is because of the above that the idea of winning whatever it takes and always being the best has been spread across youth sports. When professional baseball players perform well on the field, they are recognized on television (fame), they earn multi-million dollar contracts (wealth), and immediately after signing their contract, they are photographed for fashion magazines and hired by recognized sportswear brands to use their products (image).

The fact that children feel too pressured by their parents’ aspirations toward them and that parents following the wrong goals lead children to drop out from sports has already been proven both by previous studies [62] and by this research. It is because of the above that parents, as well as many coaches, sometimes overlook the main objectives of the game, such as the continuous learning of new skills and the values that can be instilled through sports (e.g., [63,64,65]). All this makes the child lose the desire to practice baseball (autonomy frustration); he will feel that he cannot meet his father’s expectations, for which he will feel that he is not good enough (competence frustration), and by being evaluated by others, he will believe that if he is not good at baseball he will not be appreciated by his coaches, his parents, and his teammates (relatedness frustration). A systematic review of sport dropout by Crane and Temple [66] reported that although many factors associated with sport dropout were identified, among the most important were social pressures from family and coaches. Players feel pressure to satisfy the needs of both coaches and parents, indicating that players can feel these pressures to such an extent that it contributes to their dropping out of sport. Furthermore, several studies based on the SDT [31] have shown that frustration of the needs of autonomy, competence, and relatedness were associated with greater intentions to drop out, which in turn, predicted the dropout behavior.

On the contrary, when the father stops expecting his son to become a professional player and understands that baseball is a part of the child’s personal development (personal growth), that it can be a favorable social environment for him to interact with other people and form friendships (close relationships), that fostering teamwork with others can bring a benefit to all (community contributions), and that sport is a means to be physically active and obtain health benefits (physical health), the child will have a superior motivation and will want to continue practicing baseball because he feels good doing it (autonomy satisfaction); by not being forced to win and be good at baseball, the child will not feel pressured and will feel that he can achieve goals little by little that are not based on winning (competence satisfaction), and by not being evaluated by others, he will feel that he is appreciated (relatedness satisfaction). As a result of the above, by satisfying all his three needs, the child will want to continue practicing baseball. Several studies (see [21,67] for reviews) have pointed to the important role of sport participation in satisfying the needs for autonomy, competence, and relatedness so that young people are less likely to want to drop out of the sport and have a greater intention to practice sport in the future. In the SDT [31], it is suggested that satisfaction of the three basic psychological needs promotes well-being and intention to continue being physically active, whereas the frustration of these needs, especially when coming from significant others (e.g., coaches, family, and teammates) would be more likely to negative outcomes such as ill-being and promote leaving sportive practices.

## 5. Limitations

This study has some limitations. We did not measure mother and coach expectations, and we suggest that multiple measurement of social agents would strengthen future research. The number of children in the family assessed was also not taken into account, and perhaps differences in parental expectations could be found as a function of the number of children. Another limitation is that the nature of our research was cross-sectional, so it would be appropriate to conduct a longitudinal study with the same variables to determine whether the father’s aspirations are the cause of the satisfaction or frustration of his child’s basic psychological needs and of his intention to continue or dropout of sport practice.

The socioeconomic level of the families has also not been considered given that in Mexico, the kidnapping rate is high, and this information is seen as sensitive. A low socioeconomic level could be responsible for parents having more extrinsic aspirations for their children.

Finally, only male baseball players were studied, so we cannot generalize these results to other genders and/or sports. Future research should determine the extent to which the current findings are applicable to other groups of adolescent athletes, including female and male athletes participating in a variety of sports and competitive levels.

## 6. Conclusions

The aim of this research was to gain a better understanding of how parental aspirations (from the father’s perspective) relate to the satisfaction or frustration of their children’s basic psychological needs and future intentions to play baseball (from the son’s perspective), exploring the dyadic relationship between each father and son. This study confirms that parent’s intrinsic aspirations promote their son’s sport participation and intention to remain physically active, instead of wanting to drop out, through the satisfaction of their basic psychological needs. In contrast, parent’s extrinsic aspirations increase intention to drop out from practice, rather than wanting to continue playing baseball, through the frustration of their basic psychological needs.

In light of these results, parents should consider the importance of their son’s personal growth when playing baseball, for his feeling of contributing to the improvement of society, for his building of relationships of great value to him and for his good physical health (intrinsic aspirations), which would increase the likelihood that their son would continue to want to play baseball. Conversely, parents should be aware of extrinsic aspirations for their son, such as financial success (wealth), to be recognized and admired by other people (fame), and to look attractive and wear the best sports clothes (image), which could lead to baseball dropout.

Overall, this study extends the existing sport-scientific literature by testing the impact of parental aspirations on basic psychological need satisfaction and frustration as important indicators of young baseball players’ intention to continue being physically active.

## 7. Practical Implications

From an applied perspective, this study emphasizes the importance of social agent aspirations in facilitating sport participation and preventing sport dropout, highlighting that parental aspirations have important implications for enhancing players’ intention for sport participation and reducing sport dropout. In particular, the findings suggest that interventions with parents are needed to prevent their children from dropping out of sports. For example, interventions may wish to educate parents about creating psychological environments that favor enjoyment in the activity so that their athletic children experience their sport participation in a healthy way.

## Figures and Tables

**Table 1:** Descriptive statistics and reliability analysis of all the study variables (Fathers[*n*] = 533; Sons[*n*] = 533).

Variable	Range	Mean	SD	Skewness	Kurtosis	α	CR	AVE
Fathers’ intrinsic aspirations	1–7	6.29	0.91	−2.26	5.51	0.94	0.96	0.57
Fathers’ extrinsic aspirations	1–7	3.78	1.36	−0.08	−0.96	0.94	0.94	0.51
BPN satisfaction	1–5	3.72	0.89	−1.07	0.64	0.90	0.91	0.47
BPN frustration	1–5	2.68	1.09	0.37	−1.05	0.92	0.93	0.54
Intention to continue	1–7	5.95	1.48	−1.51	1.20	0.84	0.91	0.73
Intention to drop out	1–7	1.89	1.44	1.62	1.39	0.92	0.96	0.84

*Note*. BPN = Basic psychological needs; SD = Standard deviation; α = Cronbach’s alpha; CR = Composite reliability; AVE = Average variance extracted.

**Table 2:** Matrix of bivariate correlations between the study variables.

Variable	Fathers’ Intrinsic Aspirations	Fathers’ Extrinsic Aspirations	BPN Satisfaction	BPN Frustration	Intention to Continue
Fathers’ intrinsic aspirations	-				
Fathers’ extrinsic aspirations	0.11 *	-			
BPN satisfaction	0.16 **	−0.06	-		
BPN frustration	0.03	0.32 **	−0.40 **	-	
Intention to continue	0.04	−0.17 **	0.65 **	−0.46 **	-
Intention to drop out	−0.08	0.17 **	−0.47 **	0.50 **	−0.58 **

*Note.* BPN = Basic psychological needs; ** *p* < 0.01; * *p* < 0.05.

## Data Availability

Not applicable.

## Supplementary Materials

The following are available online at https://www.mdpi.com/article/10.3390/ijerph18094587/s1, File S1: Confirmatory Factor Analysis.
