# Supplementary material for: The Father in Youth Baseball: A Self-Determination Theory Approach
Source: Int J Environ Res Public Health. 2021 Apr 26;18(9):4587. doi: 10.3390/ijerph18094587 (PMC8123654; doi:10.3390/ijerph18094587)
Supplement: Supplementary file 1 [file ijerph-18-04587-s001.zip › ijerph-1197718-supplementary.pdf]

Confirmatory factor analysis

Instruments	χ^2	*p*	*df*	χ^2/df	NNFI	CFI	RMSEA	SRMR
Aspiration Index	2240.32	< 0.001	552	4.05	0.96	0.97	0.07	0.08
Basic Psychological Need Satisfaction and Frustration Scale	835.11	< 0 .001	245	3.40	0.97	0.98	0.06	0.08
Future Intention to Practice Baseball Scale	55.22	< 0.001	19	2.91	0.99	0.99	0.06	0.03

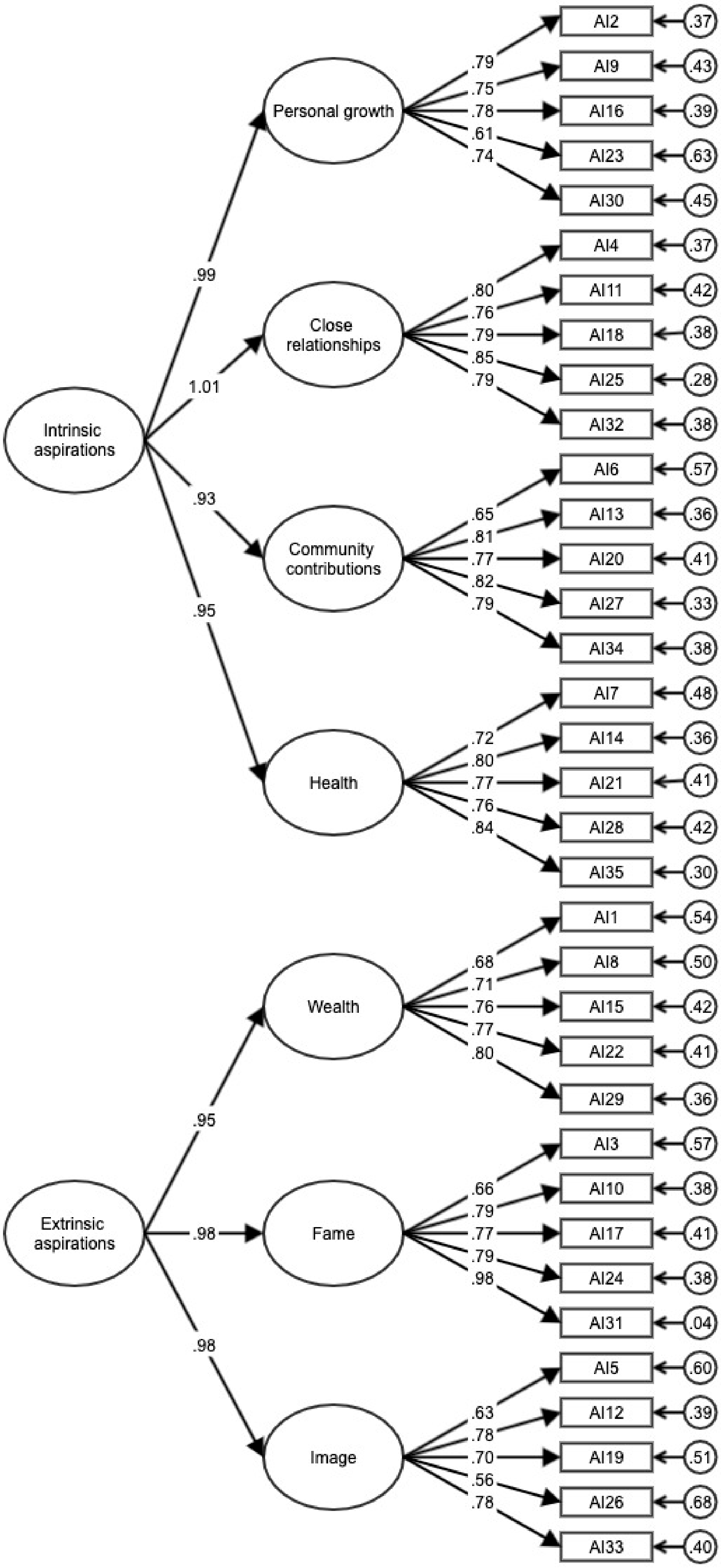

Intrinsic aspirations
Personal growth
Close relationships
Community contributions
Health
Extrinsic aspirations
Wealth
Fame
Image
.99
1.01
.93
.95
.95
.98
.98
AI2 .79 .37
AI9 .75 .43
AI16 .78 .39
AI23 .61 .63
AI30 .74 .45
AI4 .80 .37
AI11 .76 .42
AI18 .79 .38
AI25 .85 .28
AI32 .79 .38
AI6 .65 .57
AI13 .81 .36
AI20 .77 .41
AI27 .82 .33
AI34 .79 .38
AI7 .72 .48
AI14 .80 .36
AI21 .77 .41
AI28 .76 .42
AI35 .84 .30
AI1 .68 .54
AI8 .71 .50
AI15 .76 .42
AI22 .77 .41
AI29 .80 .36
AI3 .66 .57
AI10 .79 .38
AI17 .77 .41
AI24 .79 .38
AI31 .98 .04
AI5 .63 .60
AI12 .78 .39
AI19 .70 .51
AI26 .56 .68
AI33 .78 .40

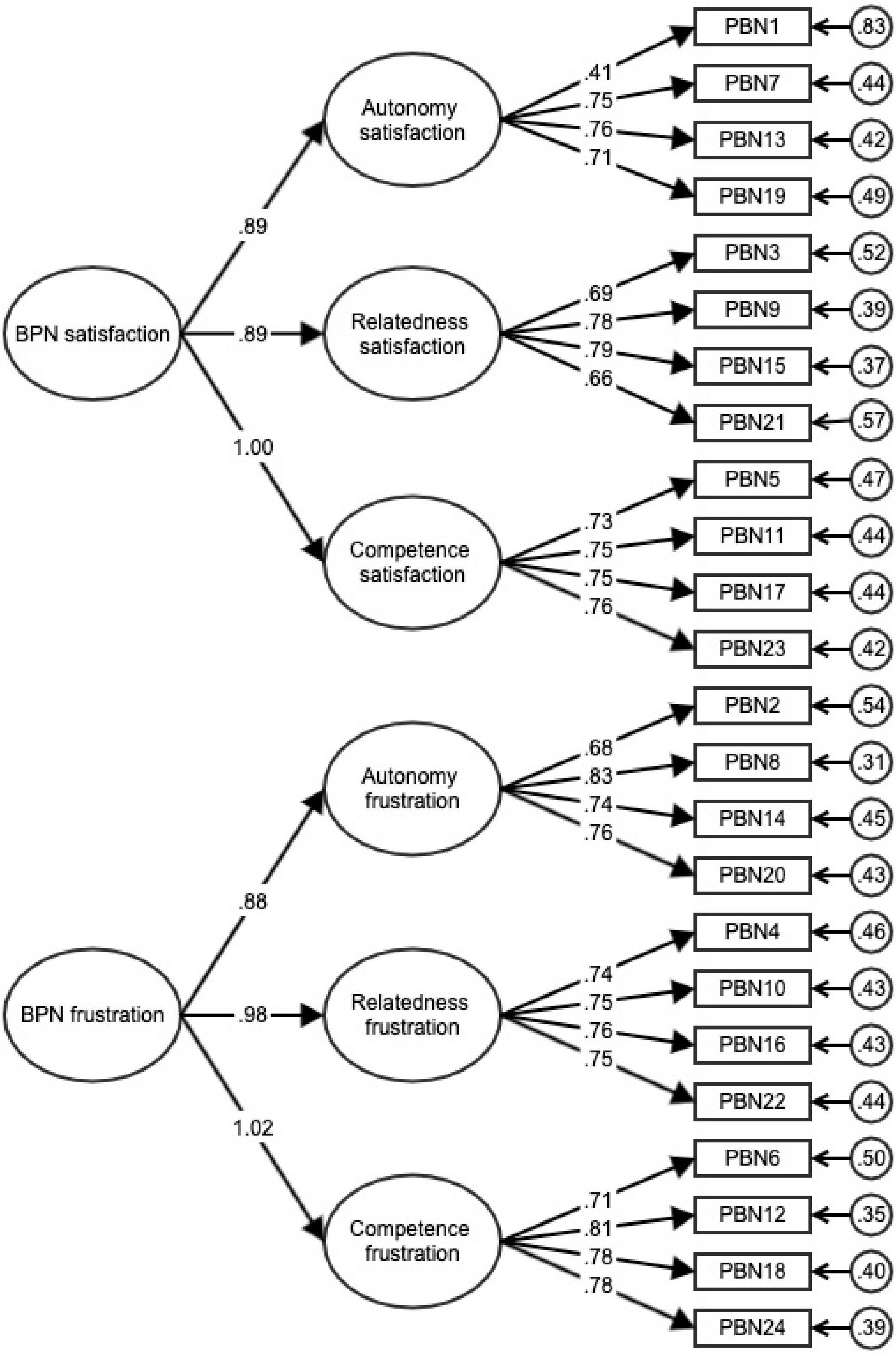
BPN satisfaction
Autonomy satisfaction
Relatedness satisfaction
Competence satisfaction
.89
.89
1.00
.41
.75
.76
.71
.69
.78
.79
.66
.73
.75
.75
.76
PBN1
PBN7
PBN13
PBN19
PBN3
PBN9
PBN15
PBN21
PBN5
PBN11
PBN17
PBN23
.83
.44
.42
.49
.52
.39
.37
.57
.47
.44
.44
.42
BPN frustration
Autonomy frustration
Relatedness frustration
Competence frustration
.88
.98
1.02
.68
.83
.74
.76
.74
.75
.76
.75
.71
.81
.78
.78
PBN2
PBN8
PBN14
PBN20
PBN4
PBN10
PBN16
PBN22
PBN6
PBN12
PBN18
PBN24
.54
.31
.45
.43
.46
.43
.43
.44
.50
.35
.40
.39

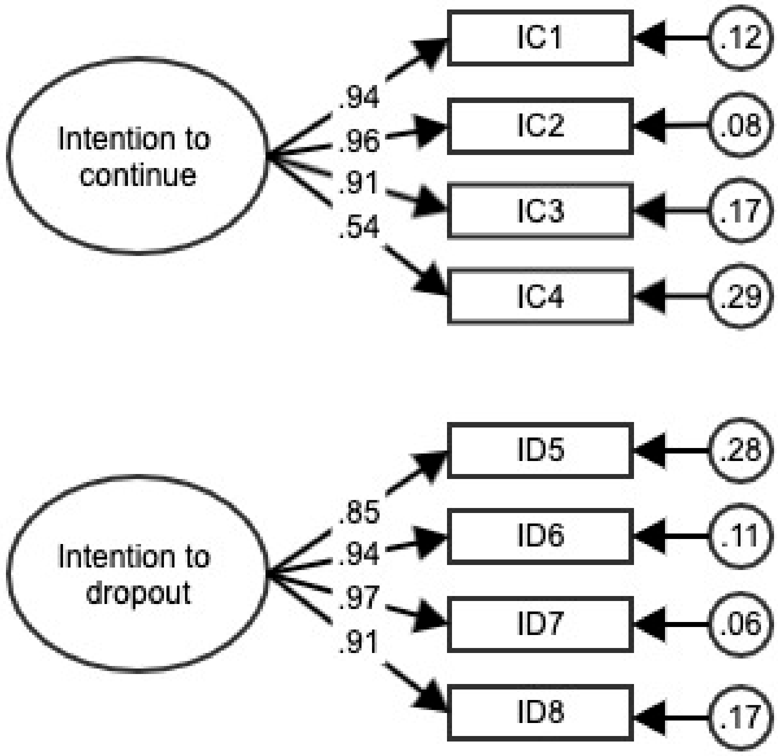
Intention to continue
IC1
IC2
IC3
IC4
.94
.96
.91
.54
.12
.08
.17
.29
Intention to dropout
ID5
ID6
ID7
ID8
.85
.94
.97
.91
.28
.11
.06
.17
